# Supplementary material for: Automated Liquid Handling Extraction and Rapid Quantification of Underivatized Amino Acids and Tryptophan Metabolites from Human Serum and Plasma Using Dual-Column U(H)PLC-MRM-MS and Its Application to Prostate Cancer Study
Source: Metabolites. 2024 Jun 30;14(7):370. doi: 10.3390/metabo14070370 (PMC11279291; doi:10.3390/metabo14070370)
Supplement: Supplementary file 1 [file metabolites-14-00370-s001.zip › metabolites-3058095-supplementary.pdf]

Automated Liquid Handling Extraction and Rapid Quantification of Underivatized Amino Acids and Tryptophan Metabolites from Human Serum and Plasma Using Dual-Column U(H)PLC-MRM-MS and Its Application to Prostate Cancer Study.

Tobias Kipura‡[1], Madlen Hotze‡[1], Alexa Hofer[1], Anna-Sophia Egger[1], Lea E. Timpen[1], Christiane A. Opitz[2], Paul A. Townsend[3,4], Lee A. Gethings[4,5], Kathrin Thedieck[1,6,7,8], Marcel Kwiatkowski*[1].

[1] Institute of Biochemistry and Center for Molecular Biosciences Innsbruck, University of Innsbruck, Innsbruck, A-6020, Austria.
[2] German Cancer Research Center (DKFZ), Heidelberg, Division of Metabolic Crosstalk in Cancer and the German Cancer Consortium (DKTK), DKFZ Core Center Heidelberg, 69120 Heidelberg, Germany
[3] Division of Cancer Sciences, Manchester Cancer Research Center, Manchester Academic Health Sciences Center, University of Manchester, Manchester, M20 4GJ, United Kingdom.
[4] School of Biosciences, Faculty of Health and Medical Sciences, University of Surrey, Guildford, Surrey, GU2 7XH, United Kingdom.
[5] Waters Corporation, Wilmslow, Cheshire, SK9 4AX, United Kingdom.
[6] Freiburg Materials Research Center FMF, Albert-Ludwigs-University of Freiburg, 79104 Freiburg, Germany.
[7] Department of Pediatrics, Section Systems Medicine of Metabolism and Signaling, University of Groningen, University Medical Center Groningen, Groningen 9700 RB, The Netherlands.
[8] Department Metabolism, Senescence and Autophagy, Research Center One Health Ruhr, University Alliance Ruhr, & University Hospital Essen, University Duisburg-Essen, 45147 Essen, Germany

‡ The authors contributed equally
* Corresponding author

E-mail: marcel.kwiatkowski@uibk.ac.at

Table of content

Supplemental Figures
Figure S1: Chromatographic separation of amino acids (AAs) and tryptophan (TRP) metabolites. P. 2
Figure S2: Chromatographic separation of the basic amino acid arginine and histidine, and the tryptophan metabolites kynurenic acid, nicotinic acid and nicotinamide extracted from human serum sample. P. 3
Figure S3: Coefficient of determination (R2) of the metabolites analyzed using dual-column U(H)PLC-MRM-MS. P. 4
Supplemental Tables
Table S1: Stable-isotope labeled canonical and non-canonical amino acids and tryptophan metabolites. P. 5
Table S2: Chromatographic parameters used for the separation of amino acids and tryptophan metabolites in the initial column screening and in the optimized method. P. 6
Table S3: Parameters used for multiple reaction monitoring (MRM) mass spectrometric analysis P. 7
Table S4: Parameters used to optimize the semi-automated extraction workflow. P. 9
Table S5: Overview of optimized pipetting settings used for semi-automated extraction of amino acids and tryptophan metabolites using the robotic liquid handling platform. P. 10
Table S6: Peak widths at half height (w1/2) of amino acids and tryptophan metabolites. P. 11
Table S7: Within-run accuracy and within-run precision. P. 12
Table S8: Between-run accuracy and between-run precision. P. 13
Table S9: Optimization of the semi-automated extraction workflow for human serum samples. P. 14
Table S10: Evaluation of optimal tip insertion depth for the transfer of extraction supernatants. P. 15
Table S11: Optimization of the semi-automated extraction workflow for human plasma samples I. P. 16
Table S12: Optimization of the semi-automated extraction workflow for human plasma samples II. P. 17
Table S13: Recovery of the optimized semi-automated workflow. P. 18
Table S14: Intra-assay variability of the optimized semi-automated workflow. P. 19
Table S15: Inter-assay precision of the optimized semi-automated workflow. P. 20
Table S16: Autosampler stability of the metabolites extracted from serum over 72 hours. P. 21
Table S17: Autosampler stability of the metabolites extracted from plasma over 72 hours. P. 22
Table S18: Quantification of amino acids and tryptophan metabolites extracted from reference plasma samples. P. 23
Table S19: Analysis of variances (ANOVA) for prostate cancer study. P. 24

Supplemental Figures

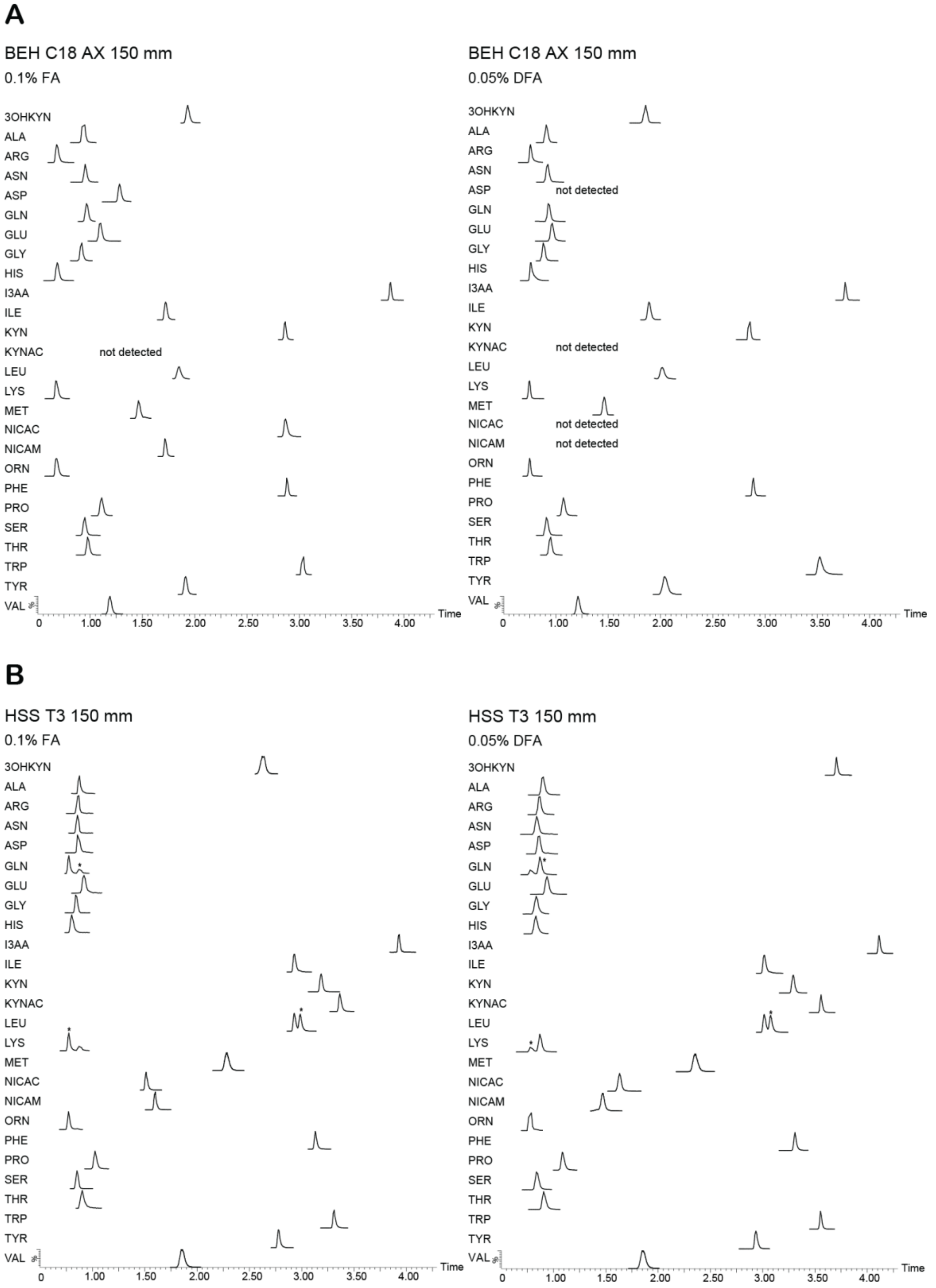

Figure S1: Chromatographic separation of amino acids (AAs) and tryptophan (TRP) metabolites. AAs and TRP metabolites were separated on a mixed-mode BEH C18 AX column (A) and a high-strength silica HSS-T3 reversed-phase column (B) using formic acid (FA) or difluoroacetic acid (DFA) as mobile phase additive. *: The MRM channels of LYS and GLN (both $[M+H]^+$: 147), and ILE and LEU (both $[M+H]^+$: 132) show two peaks due to the same intact mass selected in the first mass selective quadrupole and the same mass of the fragment selected in the second mass selective quadrupole. The peak of the indicated analyte is marked with an asterisk (*).

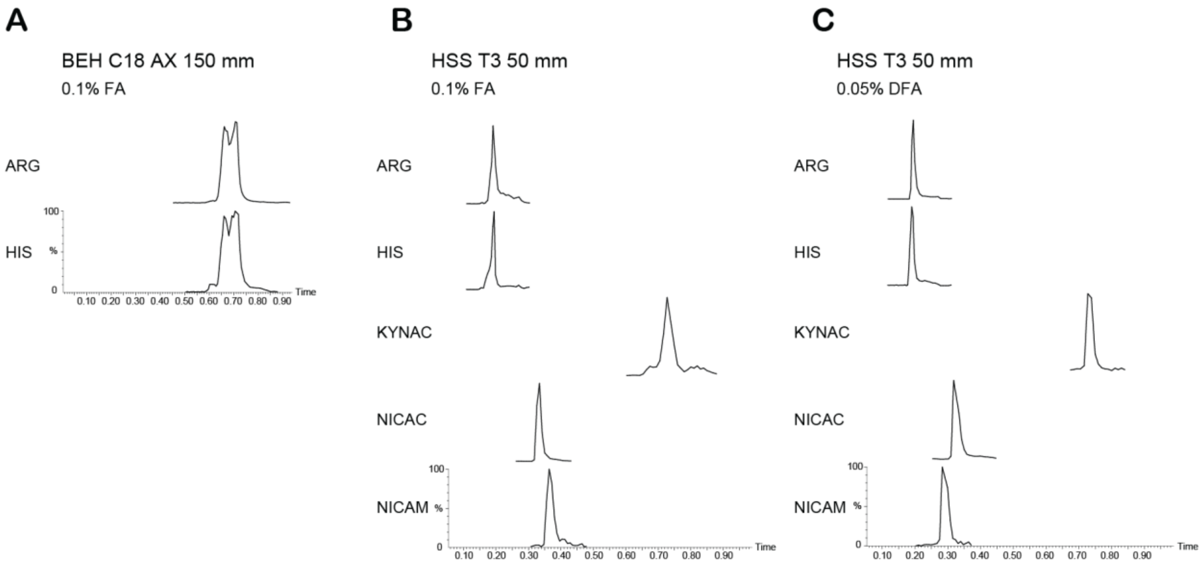

Figure S2: Chromatographic separation of the basic arginine and histidine, and the tryptophan metabolites kynurenic acid, nicotinic acid and nicotinamide extracted from human serum sample. A: Chromatograms of ARG and HIS separated on the BEH C18 AX mixed-mode column (2.1x150 mm, 1.7 µm) using 0.1% formic acid (FA) as mobile phase additive. KYNAC, NICAC and NICAM were not detected. B, C: Chromatograms of ARG, HIS, KYNAC, NICAC, NICAM separated on the HSS T3 reversed-phase column (2.1x50 mm, 1.8 µm) with 0.1% formic acid (FA) (B) or 0.05% difluoroacetic acid (DFA) (C) as mobile phase additive. ARG: Arginine, HIS: Histidine, KYNAC: Kynurenic acid, NICAC: Nicotinic acid, NICAM: Nicotinamide.

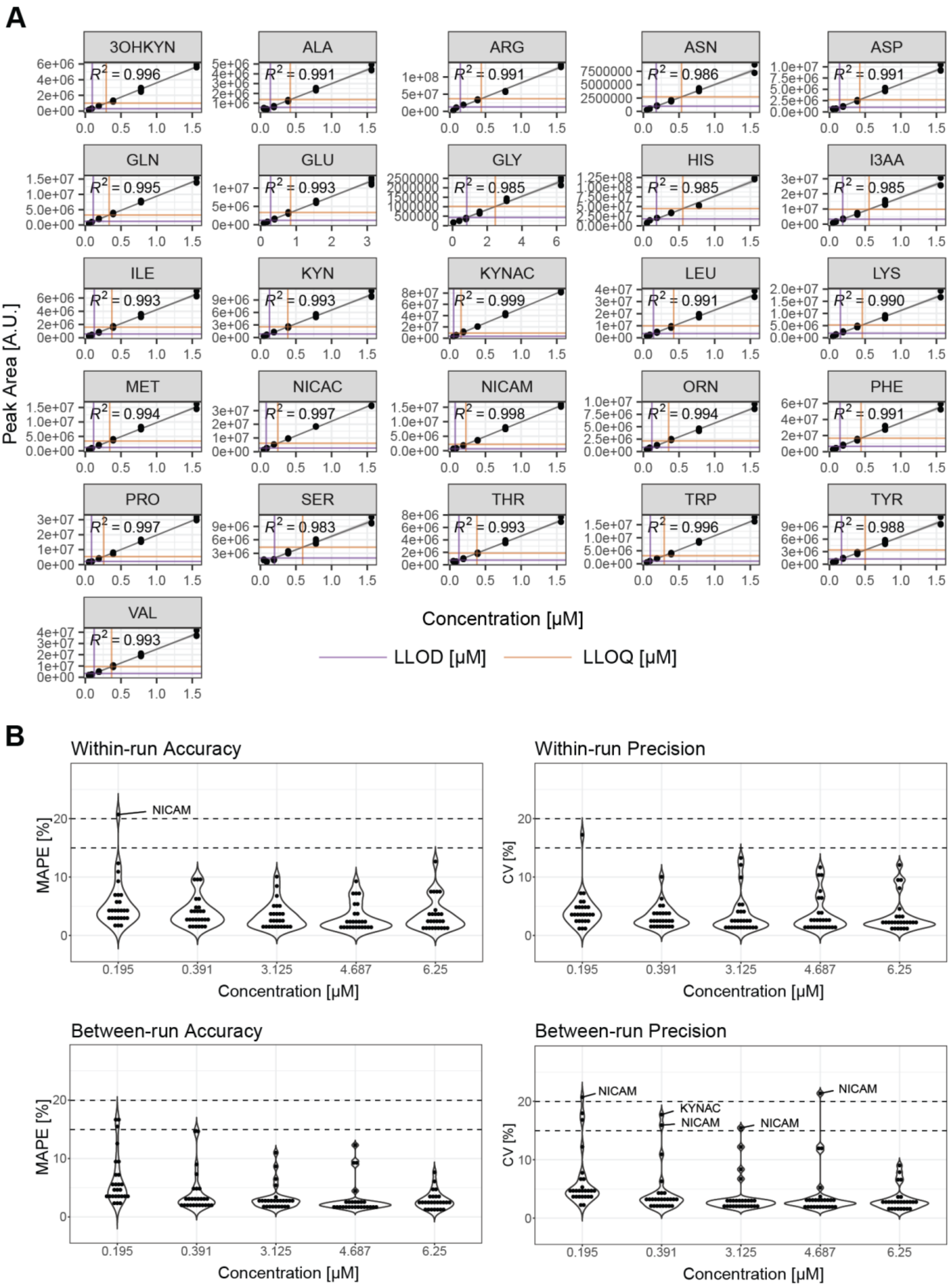

Figure S3: Quantification of amino acids and tryptophan metabolites using dual-column U(H)PLC-MRM-MS. A: Lower limit of detection (LLOD) and lower limit of quantification (LLOQ) for metabolite standards dissolved in the solvent matrix. Coefficient of determination (R2) and calculated concentrations for LLOD (purple) and LLOQ (orange) are for each metabolite (n= 4). B: Within-run (n=5) and between-run accuracy (n=20) represented by the positive amount of mean absolute percentage error (MAPE) per QC concentration, metabolites with MAPE values ≥ 20% are indicated. Within-run (n=5) and between-run (n=20) precision represented by the coefficient of variation (CV) per QC concentration, metabolites with CV values ≥ 20% at 0.195 µM or CV values ≥ 15% at all other concentrations are indicated. NICAM: Nicotinamide, KYNAC: Kynurenic acid.

Supplemental Tables

Table S1.: Stable isotope labeled canonical and non-canonical amino acids and tryptophan metabolites.

Metabolite	Abbreviation	Label, purity	Dissolved in	Stock [mM]	Vendor ID
3-Hydroxykynurenine	3OHKYN	13C3, 98%; 15N, 98%	0.1% FA	15	CNLM-10399
Alanine	ALY	13C3, 99%; 15N, 99%	0.1 N HCl	2,5	MSK-CAA-1
Arginine	ARG	13C6, 99%; 15N4, 99%	0.1 N HCl	2,5	MSK-CAA-1
Asparagine	ASN	13C4, 99%; 15N2, 99%	0.1 N HCl	2,5	MSK-CAA-1
Aspartic acid	ASP	13C4, 99%; 15N, 99%	0.1 N HCl	2,5	MSK-CAA-1
Glutamic acid	GLN	13C5, 99%; 15N, 99%	0.1 N HCl	2,5	MSK-CAA-1
Glutamine	GLU	13C5, 99%; 15N2, 99%	0.1 N HCl	2,5	MSK-CAA-1
Glycine	GLY	13C2, 99%; 15N, 99%	0.1 N HCl	2,5	MSK-CAA-1
Histidine	HIS	13C6, 97-99%; 15N3, 97-99%	0.1 N HCl	2,5	MSK-CAA-1
Indole-3-acetic acid	I3AA	13C6, 99%	50% MeOH, 0.1% FA	30	CLM-1896
Isoleucine	ILE	13C6, 99%; 15N, 99%	0.1 N HCl	2,5	MSK-CAA-1
Kynurenic acid	KYNAC	13C6, 99%	DMSO	5,1	CLM-11139
Kynurenine	KYN	13C10, 99%	0.1% FA	15	CLM-9884
Leucine	LEU	13C6, 99%; 15N, 99%	0.1 N HCl	2,5	MSK-CAA-1
Lysine	LYS	13C6, 99%; 15N2, 99%	0.1 N HCl	2,5	MSK-CAA-1
Methionine	MET	13C5, 99%; 15N, 99%	0.1 N HCl	2,5	MSK-CAA-1
Nicotinamide	NICAC	13C6, 99%	0.1% FA	15	CLM-9925
Nicotinic acid	NICAM	13C6, 99%	0.1% FA	15	CLM-9954
Ornithine	ORN	13C5, 98%	0.1% FA	2,5	MSK-NCAA-1
Phenylalanine	PHE	13C9, 99%; 15N, 99%	0.1 N HCl	2,5	MSK-CAA-1
Proline	PRO	13C5, 99%; 15N, 99%	0.1 N HCl	2,5	MSK-CAA-1
Serine	SER	13C3, 99%; 15N, 99%	0.1 N HCl	2,5	MSK-CAA-1
Threonine	THR	13C4, 97-99%; 15N, 97-99%	0.1 N HCl	2,5	MSK-CAA-1
Tryptophan	TRP	13C11, 99%; 15N2, 99%	0.1 N HCl	2,5	MSK-CAA-1
Tyrosine	TYR	13C9, 99%; 15N, 99%	0.1 N HCl	2,5	MSK-CAA-1
Valine	VAL	13C5, 99%; 15N, 99%	0.1 N HCl	2,5	MSK-CAA-1

Table S2: Chromatographic parameters used for the separation of amino acids and tryptophan metabolites in the initial column screening and in the optimized method.

**Initial Column Screening**						
**Column**	Atlantis Premier BEH C18 AX, 1.7µm, 2.1x150mm, Waters			ACQUITY UPLC HSS T3, 1.8µm, 2.1x150mm, Waters		
**Temperature Column Manager**	45°C			35°C		
**Solvents**	Eluent A: H20, 0.1% FA or 0.05% DFO, B: ACN, 0.1% FA or 0.05% DFA			Eluent A: H20, 0.1% FA or 0.05% DFA, B: ACN, 0.1% FA or 0.05% DFA		
**Gradient**	Time [min]	Flow rate [µL/min]	B [%]	Time [min]	Flow rate [µL/min]	B [%]
	0	0.350	1	0	0.450	1
	1	0.350	1	1	0.450	1
	6	0.350	70	6	0.450	70
	7	0.350	95	7	0.450	95
	8	0.350	95	8	0.450	95
	8.1	0.350	1	8.1	0.450	1
	10	0.350	1	10	0.450	1
**Optimized Method**						
**Column**	Atlantis Premier BEH C18 AX, 1.7µm, 2.1x150mm, Waters			Acquity UPLC HSS T3, 1.8µm, 2.1x50mm, Waters		
**Temperature Column Manager**	45°C			35°C		
**Solvents**	A: water, B: acetonitrile A and B containing 0.1 % FA			A: water, B: acetonitrile, A and B containing 0.05 % DFA		
**Gradient**	Time [min]	Flow rate [µL/min]	B [%]	Time [min]	Flow rate [µL/min]	B [%]
	0	0.350	1	0	0.800	1
	1	0.350	1	0.2	0.800	1
	2	0.350	20	0.8	0.800	70
	2.5	0.450	40	1	0.800	90
	4	0.450	95	1.3	0.800	90
	4.5	0.450	95	1.4	0.800	1
	4.6	0.450	1	1.9	0.800	1
	4.7	0.350	1			
	6	0.350	1			

Table S3: Parameters used for multiple reaction monitoring (MRM) mass spectrometric analysis. Cone voltage in volt [V], collision energy used for fragmentation in electron volt [eV], m/z values used for MRM transition of the analyte and stable isotope labelled internal standard (ISTD).

**Metabolite**	**Structure**	**Cone [V]**	**CE [eV]**	**MRM analyte [m/z]**	**MRM ISTD [m/z]**
3-hydroxykynurenine (3OHKYN)	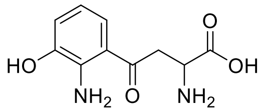	24	16	225 > 110	229 > 110
Alanine (ALA)	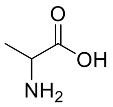	14	10	90 > 44	94 > 47
Arginine (ARG)	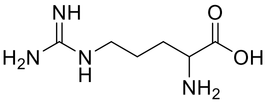	8	22	175 > 70	185 > 75
Asparagine (ASN)	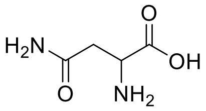	6	14	133 > 74	139 > 77
Aspartate (ASP)	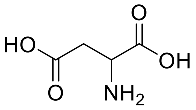	4	8	134 > 88	139 > 92
Glutamine (GLN)	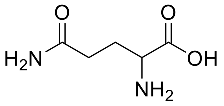	2	14	147 > 84	154 > 90
Glutamate (GLU)	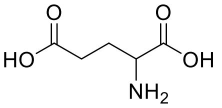	8	12	148 > 102	154 > 107
Glycine (GLY)	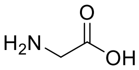	18	6	76 > 30	79 > 32
Histidine (HIS)	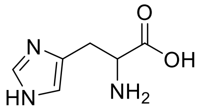	8	12	156 > 110	165 > 118
Indole-3-acetic acid (I3AA)	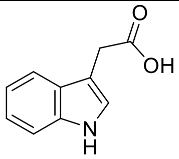	26	12	176 > 130	182 > 109
Isoleucine (ILE)	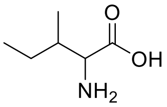	20	16	132 > 69	139 > 74
Kynurenine (KYN)	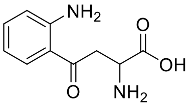	20	12	209 > 94	219 > 100
Kynurenic acid (KYNAC)	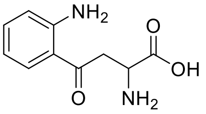	20	30	190 > 116	196 > 122

Leucine (LEU)	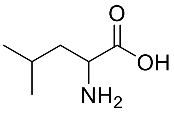	20	12	132 > 86	139 > 92
Lysine (LYS)	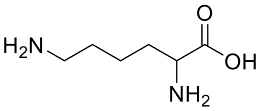	8	16	147 > 84	155 > 90
Methionine (MET)	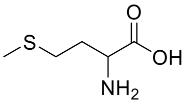	18	14	150 > 56	156 > 60
Nicotinic acid (NICAC)	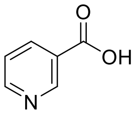	20	18	123 > 80	130 > 85
Nicotinamide (NICAM)	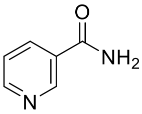	20	20	124 > 80	129 > 85
Ornithine (ORN)	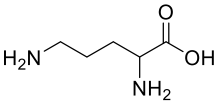	2	14	133 > 70	138 > 74
Phenylalanine (PHE)	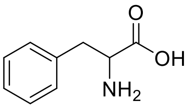	20	10	166 > 120	176 > 129
Proline (PRO)	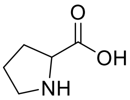	10	8	116 > 70	122 > 75
Serine (SER)	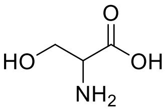	12	10	106 > 60	110 > 63
Threonine (THR)	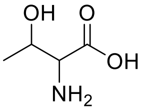	2	20	120 > 56	120 > 60
Tryptophan (TRP)	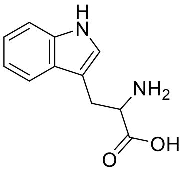	14	18	205 > 146	218 > 156
Tyrosine (TYR)	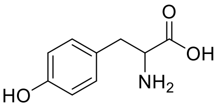	14	28	182 > 91	192 > 98
Valine (VAL)	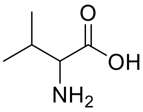	14	8	118 > 72	124 > 77

Table S4: Parameters used to optimize the semi-automated extraction workflow.

	Workflow I	Workflow II	Workflow III	Workflow IV
Sample volume [µL]	40	20	40	40
Extraction solvent volume [µL]	160	180	160	160
Incubation on the shaker at 4°C	5 min, 600 rpm	5 min, 600 rpm	5 min, 600 rpm	5 min, 600 rpm
First centrifugation, 4 °C, 4300 x g	10 min	10 min	20 min	10 min
Transfer volume of the supernatant [µL]	60	60	60	60
Volume of dilution solvent [µL]	60	60	60	60
Second centrifugation 4 °C, 4300 x g	-	-	-	10 min
Transfer volume of the supernatant [µL]	-	-	-	60
Incubation on the shaker at 4°C	5 min, 600 rpm	5 min, 600 rpm	5 min, 600 rpm	5 min, 600 rpm

Table S5: Optimized pipetting settings used for semi-automated extraction of amino acids and tryptophan metabolites using the robotic liquid handling platform.

	Pipetting Step			
Pipetting Settings	Transfer of serum to 96 well plate	Transfer of extraction solvent to 96 well plate	Transfer of 0.1% FA to 96 well plate	Transfer of supernatant to 96 well plate
Pipetting Mode	Forward	Forward	Forward	Forward
Aspiration Speed	Slow	Normal	Normal	Slow
Dispensing Speed	Normal	Normal	Normal	Normal
Air Cushion	Air bottom cushion (low viscosity aspiration)	Air bottom cushion (low viscosity aspiration)	Air bottom cushion (low viscosity aspiration)	Air bottom cushion (low viscosity aspiration)
Pipette moving speed	Normal	Normal	Normal	Normal
Tip Position Source	with respect to bottom (avoid touching the bottom)	with respect to bottom (avoid touching the bottom)	with respect to bottom (avoid touching the bottom)	with respect to bottom (avoid touching the bottom)
Customized aspiration height	no	no	no	5mm
Tip Position Destination	with respect to liquid	with respect to liquid	with respect to liquid	with respect to liquid
Customized dispensing height	no	no	no	no
Mixing	no	no	no	no
Mechanical arm motion speed	Normal	Normal	Normal	Normal

Table S6: Peak widths at half height ($w_{1/2}$) of amino acids and tryptophan metabolites. Metabolites were separated by mixed-mode chromatography (BEH C18 AX) and reversed-phase chromatography (HSS T3) using 0.1% formic acid (FA) and 0.05% difluoroacetic acid (DFA) as mobile phase modifiers.

	BEH C18 AX		HSS T3	
**Metabolite**	**0.1% FA $w_{1/2}$ [sec]**	**0.05% DFA $w_{1/2}$ [sec]**	**0.1% FA $w_{1/2}$ [sec]**	**0.05% DFA $w_{1/2}$ [sec]**
3-hydroxykynurenine (3OHKYN)	2.34	2.30	3.35	1.45
Alanine (ALA)	2.62	1.89	1.58	1.84
Arginine (ARG)	2.26	1.44	1.99	1.96
Asparagine (ASN)	1.99	2.33	2.04	2.16
Aspartic acid (ASP)	2.26	not detected	2.02	2.04
Glutamine (GLN)	2.23	2.11	2.54	2.09
Glutamic acid (GLU)	2.25	2.25	2.51	2.26
Glycine (GLY)	1.94	1.95	1.85	2.20
Histidine (HIS)	2.12	1.57	2.14	2.29
Indole-3-acetic acid (I3AA)	1.52	1.48	1.52	1.33
Isoleucine (ILE)	2.13	2.29	1.96	1.72
Kynurenine (KYN)	1.70	1.91	2.03	1.70
Kynurenic acid (KYNAC)	not detected	not detected	1.63	1.28
Leucine (LEU)	2.84	2.78	1.99	1.66
Lysine (LYS)	2.20	1.50	1.56	2.34
Methionine (MET)	2.08	2.28	2.26	2.93
Nicotinic acid (NICAC)	2.53	not detected	1.55	1.97
Nicotinamide (NICAM)	1.72	not detected	3.18	2.11
Ornithine (ORN)	2.19	1.41	1.55	2.20
Phenylalanine (PHE)	1.31	1.54	2.11	1.34
Proline (PRO)	2.28	2.12	2.09	1.82
Serine (SER)	2.14	2.17	1.78	2.20
Threonine (THR)	1.99	2.15	2.38	2.32
Tryptophan (TRP)	2.08	1.93	1.80	1.22
Tyrosine (TYR)	2.21	3.31	1.89	1.67
Valine (VAL)	2.13	2.13	2.76	2.89
Mean	2.10	2.01	2.06	1.93

Table S7: Within-run accuracy and within-run precision. Accuracy is represented by the modulus of the mean absolute percentage error (MAPE) and precision by the coefficient of variation (CV) for five concentrations. n= 5.

	0.195 µM		0.391 µM		3.125 µM		4.687 µM		6.25 µM		all concentrations	
**Metabolite**	**MAPE [%]**	**CV [%]**	**MAPE [%]**	**CV [%]**	**MAPE [%]**	**CV [%]**	**MAPE [%]**	**CV [%]**	**MAPE [%]**	**CV [%]**	**MAPE [%]**	**CV [%]**
3-hydroxykynurenine (3OHKYN)	4.71	4.33	2.73	1.25	2.51	2.98	2.62	2.69	3.82	1.84	3.28	2.62
Alanine (ALA)	4.16	4.40	3.66	4.09	1.59	1.63	2.29	2.96	3.41	3.36	3.02	3.29
Arginine (ARG)	5.97	3.44	4.10	4.23	3.42	2.32	1.97	2.30	4.11	4.54	3.91	3.37
Asparagine (ASN)	3.91	5.34	4.17	5.04	3.96	2.32	3.80	3.96	4.35	1.74	4.04	3.68
Aspartic acid (ASP)	2.96	3.48	4.63	2.51	1.61	1.09	1.46	1.78	1.14	1.45	2.36	2.06
Glutamine (GLN)	7.31	7.09	9.99	10.04	10.07	12.09	7.60	9.94	8.01	8.09	8.60	9.45
Glutamic acid (GLU)	6.55	4.88	2.94	3.35	2.99	3.72	2.73	2.96	2.21	2.99	3.48	3.58
Glycine (GLY)	9.28	7.36	4.75	6.29	5.43	5.06	6.78	6.65	7.18	9.59	6.68	6.99
Histidine (HIS)	2.98	1.18	1.02	1.18	2.65	2.26	5.40	6.21	1.65	1.80	2.74	2.53
Indole-3-acetic acid (I3AA)	2.11	2.25	3.16	2.13	2.90	1.85	2.04	2.77	1.72	2.06	2.39	2.21
Isoleucine (ILE)	2.52	2.27	1.99	2.10	1.76	1.33	1.10	1.33	1.37	2.05	1.75	1.82
Kynurenine (KYN)	5.50	1.99	1.35	1.61	3.43	2.05	1.49	0.99	1.58	2.07	2.67	1.74
Kynurenic acid (KYNAC)	12.35	17.24	9.21	3.70	4.69	5.52	5.36	7.60	7.42	9.40	7.81	8.69
Leucine (LEU)	1.35	1.44	2.21	1.70	1.26	1.60	1.17	1.70	2.83	1.53	1.76	1.59
Lysine (LYS)	3.05	3.82	3.71	3.30	3.76	4.45	2.35	2.68	2.24	2.73	3.02	3.40
Methionine (MET)	4.19	2.24	2.93	2.47	3.96	3.95	1.90	2.44	1.59	2.08	2.91	2.64
Nicotinic acid (NICAC)	3.42	3.28	9.18	5.17	8.45	13.27	7.16	10.77	7.01	1.75	7.04	6.85
Nicotinamide (NICAM)	20.71	5.78	3.21	2.84	6.77	9.93	9.27	11.66	12.66	12.07	10.52	8.46
Ornithine (ORN)	2.97	3.13	2.03	2.29	1.30	0.88	0.91	1.12	0.81	0.91	1.60	1.67
Phenylalanine (PHE)	7.06	3.59	4.85	4.27	5.09	2.19	2.09	1.77	2.04	2.49	4.23	2.86
Proline (PRO)	4.65	3.68	5.95	2.01	1.40	1.62	1.55	1.70	2.54	2.68	3.22	2.34
Serine (SER)	10.91	2.94	6.56	1.04	1.76	2.28	3.63	3.81	1.61	1.67	4.89	2.35
Threonine (THR)	3.02	4.01	2.51	2.88	1.97	1.85	1.10	1.41	3.86	2.00	2.49	2.43
Tryptophan (TRP)	3.83	5.32	1.57	1.68	2.05	1.77	1.69	1.75	3.18	0.67	2.46	2.24
Tyrosine (TYR)	1.29	0.91	2.40	2.35	1.38	0.94	1.08	1.43	0.97	0.94	1.42	1.31
Valine (VAL)	4.65	5.80	1.43	2.01	1.01	1.27	1.34	1.79	2.58	3.49	2.20	2.87
Mean	5.44	4.28	3.93	3.14	3.35	3.47	3.07	3.70	3.53	3.18	3.86	3.55

Table S8: Between-run accuracy and between-run precision. Accuracy is represented by the modulus of the mean absolute percentage error (MAPE) and precision is represented by the coefficient of variation (CV) for five concentrations. n= 20.

	0.195 µM		0.391 µM		3.125 µM		4.687 µM		6.25 µM		all concentrations	
**Metabolite**	**MAPE [%]**	**CV [%]**	**MAPE [%]**	**CV [%]**	**MAPE [%]**	**CV [%]**	**MAPE [%]**	**CV [%]**	**MAPE [%]**	**CV [%]**	**MAPE [%]**	**CV [%]**
3-hydroxykynurenine (3OHKYN)	3.09	3.17	2.10	2.57	1.28	1.66	2.37	2.83	2.02	2.28	2.17	2.50
Alanine (ALA)	9.02	5.29	4.83	4.57	3.19	2.77	1.86	2.60	3.46	2.62	4.47	3.57
Arginine (ARG)	5.41	4.90	3.04	3.72	2.89	2.69	2.42	2.80	2.77	3.25	3.31	3.47
Asparagine (ASN)	4.23	4.99	2.27	2.87	3.12	3.28	2.44	3.19	2.63	3.72	2.94	3.61
Aspartic acid (ASP)	3.75	4.26	4.38	1.59	1.85	2.23	1.60	1.56	1.61	1.54	2.64	2.24
Glutamine (GLN)	5.05	5.12	3.41	4.34	1.98	2.43	2.67	3.69	3.85	3.08	3.39	3.73
Glutamic acid (GLU)	5.36	6.61	3.12	4.03	3.00	2.97	2.07	2.30	2.30	2.75	3.17	3.73
Glycine (GLY)	15.49	16.82	5.31	6.30	5.41	6.72	4.45	5.21	4.95	6.25	7.12	8.26
Histidine (HIS)	7.56	7.75	2.45	3.13	2.88	3.23	2.71	3.30	1.53	2.06	3.43	3.89
Indole-3-acetic acid (I3AA)	3.21	4.16	1.92	2.32	3.14	2.35	2.11	2.64	2.70	2.65	2.62	2.82
Isoleucine (ILE)	2.82	3.75	2.98	3.62	1.68	1.67	2.07	2.41	0.90	1.11	2.09	2.51
Kynurenine (KYN)	4.31	4.28	2.82	3.41	2.49	2.74	2.48	2.92	2.29	2.69	2.88	3.21
Kynurenic acid (KYNAC)	12.56	17.96	14.96	17.72	8.65	12.19	9.59	11.58	7.64	9.02	10.68	13.69
Leucine (LEU)	2.61	3.16	1.60	2.00	1.69	1.92	1.23	1.43	1.44	1.75	1.71	2.05
Lysine (LYS)	1.87	2.05	1.75	1.63	2.61	2.39	1.39	1.57	1.62	1.85	1.85	1.90
Methionine (MET)	3.96	4.39	2.10	2.03	2.82	3.00	2.74	3.59	2.12	2.59	2.75	3.12
Nicotinic acid (NICAC)	9.93	12.21	8.99	10.92	6.50	8.35	8.94	12.32	6.08	7.87	8.09	10.33
Nicotinamide (NICAM)	16.63	20.71	14.35	15.91	11.00	15.45	12.29	21.36	4.50	6.90	11.75	16.07
Ornithine (ORN)	3.65	3.17	2.86	1.62	1.54	2.12	1.32	1.78	1.04	1.28	2.08	1.99
Phenylalanine (PHE)	6.87	4.58	2.06	2.70	1.93	1.64	2.11	2.67	2.70	2.46	3.13	2.81
Proline (PRO)	3.79	4.40	2.36	3.06	2.35	3.06	2.14	2.58	2.35	2.85	2.60	3.19
Serine (SER)	16.68	6.76	7.32	3.54	2.72	2.81	1.62	2.00	2.16	2.49	6.10	3.52
Threonine (THR)	5.41	4.18	3.35	3.21	2.23	2.42	1.82	2.35	3.14	1.95	3.19	2.82
Tryptophan (TRP)	5.77	4.25	2.40	3.00	2.43	2.36	1.83	2.38	3.48	2.54	3.18	2.91
Tyrosine (TYR)	3.97	2.43	1.57	1.95	1.90	1.90	1.79	2.15	1.56	1.59	2.16	2.00
Valine (VAL)	3.38	3.42	2.77	3.16	3.39	3.22	2.01	2.36	2.95	3.55	2.90	3.14
Mean	6.40	6.34	4.12	4.42	3.26	3.75	3.08	4.06	2.84	3.18	3.94	4.35

Table S9: Optimization of the semi-automated extraction workflow for human serum samples. CV: coefficient of variation (CV). For statistical analysis comparing workflow II and workflow IV, the Mann-Whitney U test was used. n=8 independent experiments.

	**Normalized metabolites CV [%]**		**Recovery [%]**	
**Metabolite**	**Workflow II**	**Workflow IV**	**Workflow II**	**Workflow IV**
Alanine (ALA)	10	12.4	72.5	88.6
Arginine (ARG)	11.5	9.1	71.5	84.1
Glutamine (GLN)	10.7	8.6	81.7	88
Glutamic acid (GLU)	17	10.1	81.7	87.6
Glycine (GLY)	18.7	10.2	71.6	93.2
Histidine (HIS)	8.4	8.8	78.3	84.3
Isoleucine (ILE)	8.6	7.7	84	82.7
Leucine (LEU)	8.2	9.1	102.9	98.9
Lysine (LYS)	15.1	11.7	72.3	84.4
Methionine (MET)	8.5	6.9	79.6	89.8
Phenylalanine (PHE)	3.5	3.1	89.5	95.4
Proline (PRO)	11.5	7.2	78.4	82.3
Serine (SER)	9.8	10.7	79.1	89.7
Threonine (THR)	30.6	32.8	26.8	68.2
Tryptophan (TRP)	7.8	6.9	77.4	86.7
Tyrosine (TYR)	9	7.7	78.5	88.7
Valine (VAL)	8.1	8.3	78.4	84.8
**Mean**	11.6	10.1	76.7	86.9
**SD**	6.1	6.2	14.9	6.6
**p-value**	0.2703		0.0006	

Table S10: Evaluation of optimal tip insertion depth for the transfer of extraction supernatants. Tip insertion depths are measured from the well bottom, values presented are CV: coefficient of variation (CV) of 3 independent experiments.

compound	3mm	4mm	5mm	6mm	7mm
Arginine (ARG)	4.2	2.7	3.4	17.4	55.7
Aspartic acid (ASP)	0.9	1.4	1.2	11.4	60.3
Glutamine (GLN)	3.9	2.1	0.5	15.3	56.1
Glutamic acid (GLU)	2.3	11.6	1.3	15.4	58.5
Glycine (GLY)	1.2	1.9	1.7	26.6	59.9
Histidine (HIS)	0.7	3.3	2.0	21.9	53.4
Isoleucine (ILE)	2.4	1.6	2.9	12.6	60.8
Leucine (LEU)	33.2	28.6	5.2	3.1	56.0
Lysine (LYS)	4.6	0.4	3.4	19.2	54.0
Methionine (MET)	0.8	2.5	2.9	11.6	60.0
Ornithine (ORN)	1.3	1.4	3.2	16.3	53.5
Phenylalanine (PHE)	13.0	1.5	17.5	25.2	66.8
Proline (PRO)	1.0	0.4	1.8	14.3	58.1
Serine (SER)	0.4	0.8	0.1	14.1	55.4
Threonine (THR)	1.9	0.2	0.8	9.3	54.3
Tryptophan (TRP)	9.7	0.8	11.1	22.3	70.8
Tyrosine (TYR)	31.7	73.0	9.1	10.4	55.3
Valine (VAL)	0.4	0.6	0.9	12.6	57.0
**Mean**	6.3	7.5	3.8	15.5	58.1
**SD**	10.1	17.7	4.5	5.9	4.6

Table S11: Optimization of the semi-automated extraction workflow for human plasma samples I. Optimizing the number of clearance steps. CV: coefficient of variation (CV). For statistical analysis comparing the workflow using one clearance step with the workflow using two clearance steps, the Mann-Whitney U test was used. n=8 independent experiments.

	**Normalized metabolites CV [%]**		**Recovery [%]**	
**Metabolite**	**One clearance step**	**Two clearance steps**	**One clearance step**	**Two clearance steps**
Alanine (ALA)	45.4	12.5	73.9	90
Arginine (ARG)	62.3	8	51.7	82.7
Asparagine (ASP)	27.1	27.2	74.9	111.8
Glutamine (GLN)	229.4	6.3	90.3	92.2
Glutamic acid (GLU)	142.2	12.9	92	90.9
Glycine (GLY)	45.5	9	78.7	89.1
Histidine (HIS)	56.6	7.5	62.5	96.1
Isoleucine (ILE)	244.7	9.6	21.9	120.2
Leucine (LEU)	113.2	14.9	52.8	109.3
Lysine (LYS)	57.2	11.4	56.9	86.1
Methionine (MET)	125.8	8	99.2	97.2
Phenylalanine (PHE)	150.4	8.5	82.1	93.3
Proline (PRO)	58.8	6.6	72.6	93.4
Serine (SER)	46.5	7.3	71	90.6
Threonine (THR)	177.7	7.1	86.3	94
Tryptophan (TRP)	58	15.9	76.5	92.2
Tyrosine (TYR)	56.9	9	26.6	112.8
Valine (VAL)	54.2	6.2	69.8	94

**Mean**	97.3	10.4	68.9	96.4
**SD**	66.6	5.1	20.8	10.2
**p-value**	<0.0001		<0.0001	

Table S12: Optimization of the semi-automated extraction workflow for human plasma samples II. Optimizing the dilution factor. CV: coefficient of variation (CV). For statistical analysis comparing the workflow using a 1:10 or a 1:20 sample dilution, Mann-Whitney U test was used. n=8 independent experiments.

	**Normalized metabolites CV [%]**		**Recovery [%]**	
**Metabolite**	**1:10 dilution**	**1:20 dilution**	**1:10 dilution**	**1:20 dilution**
3-hydroxykynurenine (3OHKYN)	11.1	22.5	117.4	111.1
Arginine (ARG)	2.7	2.9	79.4	84.2
Asparagine (ASN)	2.7	3	104.7	113.4
Aspartic acid (ASP)	2.5	3.5	106.2	108.4
Glutamine (GLN)	3.5	3.7	109.7	117.8
Glutamic acid (GLU)	3	3.5	106.3	112.9
Glycine (GLY)	2.8	4.5	110.6	118.7
Histidine (HIS)	3.3	2.4	103.5	110.7
Indole-3-acetic acid (I3AA)	2.7	2.8	112.3	124.2
Isoleucine (ILE)	2.4	2.1	108.7	119.1
Kynurenine (KYN)	3.2	4.4	114.9	117.1
Kynurenic acid (KYNAC)	35.8	38.6	105.4	200.6
Leucine (LEU)	3	2.7	109.8	117
Lysine (LYS)	2.5	3	89.4	94.7
Methionine (MET)	3.1	2.4	109.9	117.6
Phenylalanine (PHE)	3.6	2.8	108.9	115.8
Proline (PRO)	2.3	3.4	107.7	116.7
Serine (SER)	2.2	2.6	104.5	114.9
Threonine (THR)	1.8	4.5	104.9	114.9
Tryptophan (TRP)	2.3	2.6	107.8	111.7
Tyrosine (TYR)	2.4	3.3	109.9	117.7
Valine (VAL)	3.8	2.6	107.6	116.5

**Mean**	4.7	5.6	106.3	117.1
**SD**	7.2	8.5	8.0	20.5
**p-value**	0.2085		<0.0001	

Table S13: Recovery of metabolites isolated from human serum and plasma using the optimized semi-automated extraction workflow. Metabolites were extracted from human serum and human plasma. n= 7 independent experiments.

	Recovery [%]	
**Metabolite**	**Serum**	**Plasma**
3-hydroxykynurenine (3OHKYN)	98.90	92.71
Alanine (ALA)	111.27	100.74
Arginine (ARG)	93.75	87.91
Asparagine (ASN)	100.89	94.67
Aspartic acid (ASP)	98.38	89.25
Glutamine (GLN)	101.25	93.59
Glutamic acid (GLU)	98.73	91.11
Glycine (GLY)	105.28	100.08
Histidine (HIS)	111.90	98.30
Indole-3-acetic acid (I3AA)	96.80	90.54
Isoleucine (ILE)	100.99	94.57
Kynurenine (KYN)	99.38	93.31
Kynurenic acid (KYNAC)	103.22	97.64
Leucine (LEU)	101.01	94.27
Lysine (LYS)	89.83	87.06
Methionine (MET)	100.37	95.25
Nicotinic acid (NICAC)	110.14	108.71
Nicotinamide (NICAM)	105.80	97.93
Ornithine (ORN)	100.57	96.89
Phenylalanine (PHE)	100.60	94.40
Proline (PRO)	99.49	95.88
Serine (SER)	100.02	93.83
Threonine (THR)	99.52	94.46
Tryptophan (TRP)	96.08	89.68
Tyrosine (TYR)	98.07	94.85
Valine (VAL)	100.77	95.44
**Mean**	100.9	94.7
**SD**	4.9	4.5

Table S14: Intra-assay variability of the optimized semi-automated workflow. Metabolites were extracted from human serum and human plasma. CV: coefficient of variation (CV). n=7 independent experiments. Statistical analysis: Mann-Whitney U test.

	Normalized metabolites CV [%]	
**Metabolite**	**Serum**	**Plasma**
3-hydroxykynurenine (3OHKYN)	26	8.2
Alanine (ALA)	6.3	4.4
Arginine (ARG)	21.4	4.3
Asparagine (ASN)	6.6	3.4
Aspartic acid (ASP)	14.4	24.4
Glutamine (GLN)	8.1	4.1
Glutamic acid (GLU)	6.4	4.9
Glycine (GLY)	13.3	5.2
Histidine (HIS)	15.1	8.3
Indole-3-acetic acid (I3AA)	6.6	4.7
Isoleucine (ILE)	6.8	3.9
Kynurenine (KYN)	7.4	4.8
Kynurenic acid (KYNAC)	10.9	18.2
Leucine (LEU)	7	4.5
Lysine (LYS)	7.1	3.9
Methionine (MET)	6.6	2.9
Nicotinic acid (NICAC)	-	74
Nicotinamide (NICAM)	5.5	12.7
Ornithine (ORN)	7	4.5
Phenylalanine (PHE)	6.5	2.9
Proline (PRO)	6.7	3.9
Serine (SER)	7	4.2
Threonine (THR)	6.3	3.9
Tryptophan (TRP)	7	3.8
Tyrosine (TYR)	7.2	3.8
Valine (VAL)	7.9	3.7

**Mean**	9.2	8.8
**SD**	5.1	14.2

Table S15: Inter-assay precision of the optimized semi-automated workflow. Metabolites were extracted from human serum and human plasma. CV: coefficient of variation (CV). Inter-assay precision was determined for each day and over all 4 days. Statistical analysis: Mann-Whitney U test. n=7 independent experiments per day.

	**Normalized metabolites human serum CV [%]**					**Normalized metabolites human plasma CV [%]**				
**Metabolite**	**Day1**	**Day2**	**Day3**	**Day4**	**over 4 days**	**Day1**	**Day2**	**Day3**	**Day4**	**over 4 days**
3-hydroxykynurenine (3OHKYN)	7.32	10.41	24.34	11.88	15.36	10.18	-	-	2.48	-
Alanine (ALA)	4.56	2.58	2.7	2.72	7.28	2.46	2.32	1.11	1.27	2.04
Arginine (ARG)	4.81	3.51	1.64	3.4	3.8	11.47	11.47	18.76	10.29	12.21
Asparagine (ASN)	4.81	3.75	2.46	2.97	4.17	3.62	2.65	2.22	3.27	4.6
Aspartic acid (ASP)	9.09	3.2	4.44	3.63	5.57	2.38	3.39	2.22	5.21	3.77
Glutamine (GLN)	4.09	3.29	2.95	2.57	4	6.81	5.44	5.57	3.3	7.34
Glutamic acid (GLU)	9.38	4.63	9.9	8.52	12.83	4.43	8.49	7.35	7.53	8.5
Glycine (GLY)	4.37	3.66	4.99	34.15	20.01	0.12	7.68	6.84	3.08	8.1
Histidine (HIS)	3.97	4.06	2.5	3.29	4.06	3.07	3.07	6.54	7.29	4.66
Indole-3-acetic acid (I3AA)	-	-	-	-	-	5.56	4.64	5.94	5.59	8.23
Isoleucine (ILE)	4.53	3.25	4.13	3.42	4.01	1.6	1.26	2.29	1.87	2.85
Kynurenine (KYN)	5.26	3.38	2.71	2.3	4.26	1.05	2.55	1.15	5.53	2.71
Leucine (LEU)	4.53	2.54	2.03	2.53	3.64	2.87	4.44	2.29	3.1	3.3
Lysine (LYS)	4.84	3.75	8.12	6.23	13.67	1.49	3.62	1.4	1.73	2.4
Methionine (MET)	3.79	3.18	3.12	2.33	3.54	1.62	1.37	1.22	2.56	3.68
Nicotinic acid (NICAC)	51.34	83.49	62.52	8.2	86.69	1.9	1.9	12.13	-	-
Nicotinamide (NICAM)	-	-	-	-	-	-	-	18.24	4.52	-
Ornithine (ORN)	-	-	-	-	-	1.85	2.18	3.9	7.46	21.88
Phenylalanine (PHE)	4.19	3.28	2.91	2.43	3.65	0.68	3.98	1.77	0.81	3.37
Proline (PRO)	4.29	2.17	4.75	3.31	4.4	4.22	3.67	1.06	1.88	3.17
Serine (SER)	4.43	3.24	22.03	23.3	16.84	0.49	3.16	3.5	4.37	3.56
Threonine (THR)	2.78	4.38	5	4.26	5.03	1.38	2.88	1.97	2.54	3.73
Tryptophan (TRP)	4.37	3.63	3.25	3.43	3.68	3.95	4.67	4.43	1.73	3.68
Tyrosine (TYR)	4.37	2.93	4.22	3.98	3.99	1.14	0.78	0.36	4.81	3.03
Valine (VAL)	3.95	2.57	2.44	3.46	3.97	2.01	1.89	2.01	4.05	3.09
**Mean**	7.0	7.3	8.3	6.5	10.7	3.2	3.8	4.8	4.0	5.5
**SD**	10.0	17.1	13.5	7.8	17.7	2.9	2.5	5.0	2.4	4.5

Table S16: Autosampler stability of the metabolites extracted from serum over 72 hours. The extracts were incubated from 0 to 72 hours (hrs) in the autosampler at 7°C. CV: coefficient of variation [%] for each time point and over 72 hours. n= 3 independent experiments per time point.

**Metabolite**	**CV [%] metabolites from serum**				**CV [%] over 72 hrs**	**Decrease/ Increase [%]**
	**0 hrs**	**24 hrs**	**48 hrs**	**72 hrs**		
Arginine (ARG)	0.04	2.84	2.9	4.35	3.37	0.57
Aspartic acid (ASP)	2.17	2.25	5.55	5.47	4.31	4.93
Glutamine (GLN)	0.94	2.87	3.43	3.77	2.69	0.98
Glutamic acid (GLU)	0.68	4.21	4.49	4.17	3.66	2.02
Glycine (GLY)	0.18	13.89	4.77	17.37	11.22	-5.63
Histidine (HIS)	0.38	1.28	1.99	5.14	2.75	0.24
Isoleucine) ILE	2.83	0.84	6.15	4.85	3.83	4.38
Leucine (LEU)	2.83	37.35	25.74	7.32	20.42	10.59
Lysine (LYS)	3.2	3.04	0.77	4.94	3.52	0.50
Methionine (MET)	1.95	2.16	5.32	5.07	3.91	4.81
Ornithine (ORN)	1.63	2.63	2.91	8.61	4.79	-0.07
Phenylalanine (PHE)	3.38	6.33	11.97	7.37	6.51	-0.05
Proline (PRO)	3.05	6.18	4.21	4.48	4.7	2.43
Serine (SER)	2.87	5.52	5.01	10.75	6.51	-0.58
Threonine (THR)	1.24	5.6	4.63	9.55	6.06	-2.07
Tryptophan (TRP)	6.31	7.16	4.68	7.93	6.24	4.26
Tyrosine (TYR)	6.33	53.61	75.34	0.1	35.7	21.01
Valine (VAL)	1.21	1.62	4.13	4.21	3.21	3.94
**Mean**	2.3	8.9	9.7	6.4	7.4	2.9
**SD**	1.8	13.9	17.3	3.7	8.2	5.7

Table S17: Autosampler stability of the metabolites extracted from plasma over 72 hours. The extracts were incubated from 0 to 72 hours (hrs) in the autosampler at 7°C. CV: coefficient of variation [%] for each time point and over 72 hours. n= 3 independent experiments per time point.

Metabolite	CV [%] metabolites from plasma				CV [%] over 72 hrs	Decrease/ Increase [%]
	0 hrs	24 hrs	48 hrs	72 hrs		
3-hydroxykynurenine (3OHKYN)	0.62	1.4	2.74	13.29	6.36	-4.10
Alanine (ALA)	1.53	1.11	2.7	1.7	2.98	0.94
Arginine (ARG)	4.88	19.76	4.62	10.94	10.74	-5.36
Asparagine (ASN)	2.09	1.89	0.04	1.4	2.62	1.63
Aspartic acid (ASP)	1.93	0.44	0.92	1.42	3.65	4.30
Glutamine (GLN)	1.02	1.43	0.61	2.08	2.72	0.49
Glutamic acid (GLU)	1.18	0.92	0.12	1.35	2.46	2.64
Glycine (GLY)	3.12	3.14	10.07	1.13	7.06	-1.26
Histidine (HIS)	0.67	13.98	6.46	11.22	8.75	-7.82
Indole-3-acetic acid (I3AA)	1.09	0.79	1.11	2.08	2.11	-0.09
Isoleucine (ILE)	2.29	1.06	1.42	2.28	2.99	3.30
Kynurenine (KYN)	2.04	1.29	4.31	12.9	6.44	-5.16
Leucine (LEU)	1.56	1.31	1.22	1.83	2.94	4.07
Lysine (LYS)	1.37	0.15	0.84	2.08	1.58	-1.73
Methionine (MET)	1.79	1.24	0.22	1.48	3.01	4.37
Nicotinic acid (NICAC)	3.49	2.93	8.72	6.31	5.7	-1.99
Nicotinamide (NICAM)	2.25	3.5	0.52	7.95	4.75	-0.89
Ornithine (ORN)	1.37	2.73	0.02	2.43	2	-1.43
Phenylalanine (PHE)	1.11	1.53	1.17	2.05	2.92	5.53
Proline (PRO)	1.65	1.86	1.12	1.91	2.29	2.73
Serine (SER)	0.75	2	0.78	0.36	2.86	3.01
Threonine (THR)	2.17	5.37	1.44	2.06	4.66	0.85
Tryptophan (TRP)	1.77	1.82	0.54	1.11	2.65	3.70
Tyrosine (TYR)	0.55	0.4	1.03	2.5	2.36	3.57
Valine (VAL)	2.25	1.3	0.77	2.15	2.53	2.02

**Mean**	1.8	2.9	2.1	3.8	4.0	0.5
**SD**	1.0	4.4	2.7	4.0	2.3	3.5

Table S18: Quantification of amino acids and tryptophan metabolites extracted from reference plasma sample. The quantified metabolite levels were compared with the certified values of the NIST SRM 1950 reference sample (*). Mean absolute error (MAE) was calculated as the average of the subtraction of each individual calculated concentration (n= 7 independent experiments) from the provided reference value/certified value. Mean absolute percentage error (MAPE) was calculated as the average of the subtraction of the reference value/certified value from each individual calculated concentration (n=7 independent experiments) divided by the calculated concentration as described by Gray et al [1]. Official Certificate of Analysis is available at www.nist.gov.

Compound	NIST SRM 1950 Reference Values, (*) Certified Values [µM]	Absolute Quantification median ± SD [µM]	Absolute Quantification mean ± SD [µM]	CV [%]	MAE [µM]	MAPE [%]
Alanine (ALA)	300 ± 26	259.19 ± 7.58	258.3 ± 7.58	2.93	41.7	16.2
Arginine (ARG)	81.4 ± 2.3 (*)	98.09 ± 8.43	101.72 ± 8.43	8.29	20.3	19.6
Asparagine (ASN)	-	33.58 ± 2.1	33.4 ± 2.1	6.29	-	-
Aspartic acid (ASP)	-	12.87 ± 2.9	13.6 ± 2.9	21.35	-	-
Glutamine (GLN)	-	401.79 ± 12.72	407.01 ± 12.72	3.13	-	-
Glutamic acid (GLU)	67.4 ± 18 (*)	103.28 ± 8.34	100.62 ± 8.34	8.29	33.2	32.6
Glycine (GLY)	245 ± 16	263.92 ± 29.28	277.18 ± 29.28	10.56	32.2	10.8
Histidine (HIS)	72.6 ± 3.6	74.96 ± 3.9	73.95 ± 3.9	5.27	1.4	1.6
Indole-3-acetic acid (I3AA)		1.01 ± 0.06	1.03 ± 0.06	5.41	-	-
Isoleucine (ILE)	55.5 ± 3.4	61.89 ± 2.7	61.71 ± 2.7	4.38	6.2	9.9
Kynurenine (KYN)	-	0.93 ± 0.07	0.95 ± 0.07	6.99	-	-
Kynurenic acid (KYNAC)	-	0.05 ± 0.01	0.05 ± 0.01	11.37	-	-
Leucine (LEU)	100 ± 6	111.91 ± 3.64	112.93 ± 3.64	3.22	12.5	11.0
Lysine (LYS)	140 ± 14	139.47 ± 5.06	138.75 ± 5.06	3.65	1.3	1.0
Methionine (MET)	22.3 ± 1.8	22.89 ± 0.74	22.91 ± 0.74	3.22	0.6	2.6
Ornithine (ORN)	52.1 ± 2.8 (*)	57.96 ± 2.5	58.56 ± 2.5	4.27	6.5	10.9
Phenylalanine (PHE)	50.8 ± 7 (*)	58.62 ± 2.8	59.35 ± 2.8	4.72	8.4	13.9
Proline (PRO)	177 ± 9	189.71 ± 5.54	189.78 ± 5.54	2.92	12.8	6.7
Serine (SER)	95.9 ± 4.3	103.13 ± 5.33	104.89 ± 5.33	5.08	9.0	8.4
Threonine (THR)	119 ± 6	118.49 ± 4.64	119.09 ± 4.64	3.89	0.4	0.5
Tryptophan (TRP)	-	41.75 ± 1.45	42.03 ± 1.45	3.44	-	-
Tyrosine (TYR)	57.3 ± 3	58.6 ± 1.96	58.17 ± 1.96	3.37	0.9	1.4
Valine (VAL)	182 ± 10	179.54 ± 4.55	180.64 ± 4.55	2.52	1.6	0.9
Mean				5.8	11.8	9.25

1. Gray, N.; Zia, R.; King, A.; Patel, V.C.; Wendon, J.; McPhail, M.J.; Coen, M.; Plumb, R.S.; Wilson, I.D.; Nicholson, J.K. High-Speed Quantitative UPLC-MS Analysis of Multiple Amines in Human Plasma and Serum via Precolumn Derivatization with 6-Aminoquinolyl-N-hydroxysuccinimidyl Carbamate: Application to Acetaminophen-Induced Liver Failure. *Anal. Chem.* **2017**, *89*, 2478–2487. https://doi.org/10.1021/acs.analchem.6b04623.

Table S19: Analysis of variances (ANOVA) for prostate cancer study. p-values per metabolite determined by ANOVA including all patient conditions and controls.

**Metabolite**	**p-value**
Alanine (ALA)	0.1020
Arginine (ARG)	0.2297
Asparagine (ASN)	0.1887
Aspartic acid (ASP)	0.3752
Glutamine (GLN)	0.9975
Glutamic acid (GLU)	0.8591
Glycine (GLY)	0.3847
Histidine (HIS)	0.8924
Isoleucine (ILE)	0.3837
Leucine (LEU)	0.1868
Lysine (LYS)	0.1825
Methionine (MET)	0.0489
Ornithine (ORN)	0.0260
Phenylalanine (PHE)	0.0827
Proline (PRO)	0.5386
Serine (SER)	0.1226
Threonine (THR)	0.8198
Tryptophan (TRP)	0.1831
Tyrosine (TYR)	0.0223
Valine (VAL)	0.3907
